# *Porphyromonas gingivalis* interaction with *Candida albicans* allows for aerobic escape, virulence and adherence

**DOI:** 10.1016/j.bioflm.2023.100172

**Published:** 2023-12-17

**Authors:** Caroline A. de Jongh, Floris J. Bikker, Teun J. de Vries, Arie Werner, Susan Gibbs, Bastiaan P. Krom

**Affiliations:** a Department of Preventive Dentistry, Academic Centre for Dentistry Amsterdam (ACTA), University of Amsterdam and Vrije Universiteit Amsterdam, Amsterdam, the Netherlands; b Department of Oral Biochemistry, Academic Centre for Dentistry Amsterdam (ACTA), University of Amsterdam and Vrije Universiteit Amsterdam, Amsterdam, the Netherlands; c Department of Periodontology, Academic Centre for Dentistry Amsterdam (ACTA), University of Amsterdam and Vrije Universiteit Amsterdam, Amsterdam, the Netherlands; d Department of Dental Materials Science, Academic Centre for Dentistry Amsterdam (ACTA), University of Amsterdam and Vrije Universiteit Amsterdam, Amsterdam, the Netherlands; e Department of Oral Cell Biology, Academic Centre for Dentistry Amsterdam (ACTA), University of Amsterdam and Vrije Universiteit Amsterdam, Amsterdam, the Netherlands; f Department of Molecular Cell Biology and Immunology, Amsterdam UMC, Vrije Universiteit Amsterdam, Amsterdam, the Netherlands

**Keywords:** *Porphyromonas gingivalis*, *Candida albicans*, Survival, Adherence, Gingipains

## Abstract

In the oral cavity *Candida albicans* interacts with many oral bacteria, including *Porphyromonas gingivalis*, both physically and metabolically. The aim of this *in vitro* study was to characterize these interactions and study their effects on the survival of *P. gingivalis*. First, metabolic interactions were evaluated by counting the colony forming units (CFU) after co-culturing. The results indicated that the anaerobic bacterium *P. gingivalis* survives under aerobic conditions when co-cultured with *C. albicans*. This is due to the oxygen consumption by *C. albicans* as determined by a reduction in survival upon the addition of Antimycin A. By measuring the protease activity, it was found that the presence of *C. albicans* induced gingipain activity by *P. gingivalis*, which is an important virulence factor. Adherence of *P. gingivalis* to hyphae of *C. albicans* was observed with a dynamic flow system. Using various *C. albicans* mutants, it was shown that the mechanism of adhesion was mediated by the cell wall adhesins, members of the agglutinin-like sequence (Als) family: Als3 and Als1. Furthermore, the two microorganisms could be co-cultured into forming a biofilm in which *P. gingivalis* can survive under aerobic culturing conditions, which was imaged using scanning electron microscopy. This study has further elucidated mechanisms of interaction, virulence acquisition and survival of *P. gingivalis* when co-cultured with *C. albicans*. Such survival could be essential for the pathogenicity of *P. gingivalis* in the oxygen-rich niches of the oral cavity. This study has emphasized the importance of interaction between different microbes in promoting survival, virulence and attachment of pathogens, which could be essential in facilitating penetration into the environment of the host.

## 1 Introduction

Over 700 different species of bacteria, fungi, viruses and protozoa have been discovered in the oral cavity [1]. These species interact with each other in various communities, collectively known as the oral microbiota. In a healthy environment, the oral microbiome is in balance with its host. However, dysbiosis occurs when pathogenic microorganisms increase in relative number due to a change in the oral environment of the host [2]. Factors that are associated with oral dysbiosis include poor oral hygiene, smoking, diet, genetics and impaired salivary function [3]. This can result in oral diseases such as caries, gingivitis and periodontitis. The bacterium most often associated with periodontitis is *Porphyromonas gingivalis* [4].

*P. gingivalis* is a strictly anaerobic, Gram-negative bacterium [4,5]. In addition to periodontitis, *P. gingivalis* has been associated with systemic diseases such as atherosclerosis, rheumatoid arthritis and Alzheimer's disease, where *P. gingivalis* was detected in the arterial wall, synovium and brain, respectively [[6], [7], [8]]. One of the major virulence factors of *P. gingivalis* are the gingipains [9]. These proteases are either specific for arginine (Rgp) or lysine (Kgp). They aid in the survival of *P. gingivalis* within the host, but also increase its pathogenicity by degrading host extracellular matrix and adhesion molecules that are essential for the integrity of host tissue barriers; and degrading cytokines that are required for the host immune response. Gingipains can be secreted, expressed on the surface of the bacterium, or released by outer membrane vesicles into the surrounding microenvironment [10].

As *P. gingivalis* has been associated with various systemic diseases, it needs to translocate the oral mucosa and other tissue barriers. One of these mechanisms could be via the interaction with other microorganisms that are present in the oral cavity [11]. *Candida albicans* is commonly found in the oral cavity of healthy individuals and is known to interact with various bacteria [12]. It is a polymorphic yeast, meaning it has various morphologies depending on environmental conditions. In the oral cavity of healthy individuals, *C. albicans* is commonly present in its yeast morphology (oval-shaped unicellular fungus). However, *C. albicans* is an opportunistic pathogen and when the immune system of the host is impaired the fungus can increase in relative numbers and form hyphae that have the ability to invade the mucosal tissue of the oral cavity. This results in oropharyngeal candidiasis, more commonly known as oral thrush [13]. *C. albicans* has been observed to interact with various bacterial species, which can occur at different levels [14]. In metabolic interactions, *Candida*-mediated oxygen removal is important. This could create a niche for *P. gingivalis* to survive in the oral cavity [15]. In physical interactions, research has shown that various oral bacteria will adhere to the hyphae of *C. albicans* [14,16]. These physical interactions commonly involve the hyphae-associated proteins: specific surface proteins that are expressed on the hyphae of *C. albicans*, different than when it is in yeast form. Examples of these proteins are members of the agglutinin-like sequence (Als) family Als1 and Als3, Hyphal wall protein 1 (Hwp1) and Cell wall adhesion protein 1 (Eap1). All these proteins are specifically expressed on the cell wall of the hyphae and are involved in adhesion of *C. albicans* to biotic and a-biotic surfaces [17]. Both metabolic and physical interactions between *P. gingivalis* and *C. albicans* may increase the ability of *P. gingivalis* to invade the host.

The aim of the current research was to characterize the interaction between *P. gingivalis* and *C. albicans* and to study its effect on the survival of *P. gingivalis*. First, in a planktonic (free-living) culture it was found that *P. gingivalis* was able to survive in the presence of *C. albicans*, even when cultured aerobically. Second, in a dynamic flow system *P. gingivalis* was shown to adhere to the hyphae of *C. albicans* and an insight is given into the molecular mechanism of adhesion. And lastly, images of a mixed-species biofilm with both *P. gingivalis* and *C. albicans* show the physical interactions of these two microorganisms more clearly.

## 2 Materials and methods

### 2.1 Strains and growth conditions

For this research, two different strains of *P. gingivalis* and *C. albicans* were used, a high virulent and a low virulent strain for each microorganism. *P. gingivalis* W83 is more virulent than ATCC 33277. W83 has a K1 type polysaccharide capsule and 33277 has no capsule [18,19]. The strains have different major fimbriae: *P. gingivalis* ATCC 332777 has minor fimbriae, unlike *P. gingivalis* W83 [20]. *C. albicans* SC5314 is more virulent than ATCC 10231, as SC5314 grows longer hyphae that can invade tissue structures, while ATCC 10231 is non-invasive [21].

*P. gingivalis* W83 and ATCC 33277 were grown on Anaerobic Blood Agar (ABA), which consists of Tryptic Soy Agar (TSA, Becton Dickinson, Franklin Lakes, USA) supplemented with 5 % defibrinated sheep blood (Biotrading, Mijdrecht, the Netherlands), 2 mg/mL glucose (Merck), 5 μg/mL hemin (H, Sigma-Aldrich, St. Louis, USA) and 1 μg/mL menadione (M; Vitamin K, Sigma-Aldrich). Pre-cultures were inoculated overnight in Brain Heart Infusion (BHI, Becton Dickinson) supplemented with H/M. *P. gingivalis* cultures were incubated at 37 °C under anaerobic conditions: 10 % H_2_, 10 % CO_2_, 80 % N_2_.

*C. albicans* was grown on TSA at 30 °C. Pre-cultures were inoculated overnight in BHI at 30 °C, under aerobic conditions, while shaking at 150 rpm. All microorganisms used are listed in Table 1.

**Table 1:** Microorganisms used in this study.

Strain name	Characteristics/Genotype	Reference
***P. gingivalis* strains**		
W83	Wild type, Capsular serotype 1 (K1, thick capsule)	[22]
ATCC 33277	Wild type, Non-encapsulated (K−)	
***C. albicans* strains**		
SC5314	Wild type	[23]
ATCC 10231	Wild type	
*als1* Δ/Δ	SC5314 *als1-1Δ::FRT/als1-2Δ::FRT*	[16]
*als3* Δ/Δ	SC5314 *als3-1Δ::FRT/als3-2Δ::FRT*	[16]
*als1 als3* ΔΔ/ΔΔ	SC5314 *als1-1Δ::FRT/als1-2Δ::FRT als3-1Δ::FRT/als3-2Δ::FRT*	[16]
*hwp1* Δ/Δ	*ura3/ura3 hwp1/hwp1-URA3*	[24]
*eap1* Δ/Δ	*ura3::λimm434/ura3::λimm434 his1::hisG/his1::hisG arg4::hisG/arg4::hisG eap1::URA3/eap1::ARG4*	[25]

### 2.2 Planktonic co-culture of *P. gingivalis* and *C. albicans*

*P. gingivalis* W83 and ATCC 33277 and *C. albicans* SC5314 pre-cultures were diluted in BHI + H/M to a final optical density measured at 600 nm (OD_600_) of 0.01 and 0.1, respectively. These OD_600_ values equate to approximately 10^8^ colony forming units (CFU) per mL for *P. gingivalis* and 10^6^ CFU/mL for *C. albicans.* Heat-killing of *C. albicans* was performed at 80 °C for 15 min and Antimycin A (Sigma-Aldrich) was added to a final concentration of 10 μM. Antimycin A is an inhibitor of the classical respiratory pathway in mitochondria of *C. albicans* and blocks the majority of mitochondrial oxygen consumption [26]. The cultures were incubated statically at 37 °C, either aerobically or anaerobically. Before incubation and after 48 h, 10 μL samples were taken from each culture and a serial dilution was made. 10 μL of each dilution was spotted on ABA plates and incubated anaerobically for 5–7 days for CFU counting. The CFU were detectable if there were 5 of more CFU per droplet (equating to 500 CFU/mL). If there were less than 5 CFU present, it was determined to be under the detection limit.

### 2.3 Determination of gingipain activity

To determine the gingipain activity an assay was performed using gingipain-specific fluorogenic substrates, BikKam 14, specific for Kgp, and BikKam 16, specific for Rgp, essentially as described elsewhere [27]. In short, *P. gingivalis* and *C. albicans* were co-cultured as described above. After 48 h, the samples were centrifuged for 1 min at 21300×*g* and the supernatant was collected. 50 μL of each supernatant sample was added in black clear-bottom 96-well plates (Greiner, Alphen aan den Rijn, the Netherlands). The fluorogenic substrates were diluted in Tris buffered saline (TBS) and 50 μL was added to the samples with a final concentration of 16 μM. Plates were read for 2 h at 37 °C, with 1 min intervals on a microplate reader (Spectramax M2, Molecular Devices, San Jose, USA) at an excitation wavelength of 485 nm and emission wavelength of 530 nm. Protease activity was determined by calculating the relative fluorescence per minute (RF/min) of the initial linear part of the curve that resulted from the kinetic measurement, as described elsewhere [27]. If a sample showed an RF/min value of 5 or higher, it was considered positive for gingipain activity.

### 2.4 Real-time analysis of *P. gingivalis* adhesion to *C. albicans*

The Bioflux Z1000 setup is as described previously [16]. Briefly, the channels of 48-well microfluidics plates (Fluxion Biosciences, Alameda, USA) were coated for 30 min with pre-warmed 10 % fetal bovine serum (FBS, Sigma-Aldrich) in phosphate buffered saline (PBS) using a flow rate of 0.5 dyne/cm^2^. *C. albicans* pre-cultures were diluted to an OD_600_ of 0.25 in Yeast Nitrogen Base pH 7.0 supplemented with 0.5 % glucose (YNB, Becton Dickinson). *C. albicans* suspensions were added to the outlet well of the microfluidics plates and with a flow of 0.5 dyne/cm^2^ the channels were filled with cells. The flow was stopped for 30 min to allow the *C. albicans* to adhere to the surface and afterwards additional YNB was added to the inlet well and flowed at a rate of 0.5 dyne/cm^2^ for 2.5 h to allow hyphae formation. *P. gingivalis* W83 and ATCC 33277 pre-cultures were diluted to an OD_600_ of 0.2 and centrifuged for 1 min at 21300×*g*. They were stained with a 5 nM solution of carboxyfluorescein succinimidyl ester (CFSE, Sigma-Aldrich) in PBS for 30 min at 37 °C. After staining, the cells were washed twice with PBS. The CFSE-labeled *P. gingivalis* were added to the inlet wells after hyphae were formed and flowed in the channel at a rate of 0.5 dyne/cm^2^. Images were acquired at three random positions with hyphae in the channel every 5 min for 2 h, using a Axio Observer Zeiss Z1 microscope (Zeiss, Oberkochen, Germany). Using a 20× objective, brightfield and green fluorescent illumination (Excitation filter: 405/30 Emission filter: 520/40) were used to visualize *C. albicans* and CFSE-labeled *P. gingivalis*, respectively. Image analysis was performed using ImageJ (version 1.53e) [28]. Per experiment, each condition was performed in duplicate and three positions were imaged per channel, resulting in 6 time-lapses per condition. All results are based on the average of each of the 6 time-lapses in three independent experiments. After 1 h, the total number of hyphae in the image were counted and the number of hyphae that had visible fluorescent *P. gingivalis* attached was assessed. Then, of these hyphae that had fluorescence, the relative number of *P. gingivalis* was determined by digitally drawing the outline of the hyphae as a region of interest (ROI) and quantifying the mean fluorescence using ImageJ (version 1.53e). The fluorescent intensity value of the background was subtracted to correct for the fluorescent *P. gingivalis* that are not adhering to the hyphae.

### 2.5 Mixed-species biofilm formation and scanning electron microscopy (SEM)

For the acquisition of SEM images, the biofilms were grown on polystyrene squares cut from a 24-well cell culture plate. The surfaces were sterilized using 80 % ethanol, air-dried and placed in a sterile 24-well plate. Pre-cultures of *P. gingivalis* and *C. albicans* were diluted in 10 % BHI + H/M to an OD_600_ of 0.2 and 0.1, respectively. *C. albicans* suspensions were added and was allowed to adhere for 90 min at 37 °C. After adherence, the polystyrene surfaces were washed with PBS and the *P. gingivalis* suspension was added. The surfaces were incubated at 37 °C on a shaking plate for 1 h. The surfaces were washed with PBS again, before adding 10 % BHI + H/M medium. The plates were incubated aerobically at 37 °C for 48 h, with a medium change after 24 h. After incubation, the biofilms were processed for scanning electron microscopy (SEM) analysis, as follows: Samples were washed with PBS and fixed in 2 % glutaraldehyde (Sigma-Aldrich) overnight. The samples were then dehydrated with an ethanol series (35 %, 50 %, 75 %, 95 %, 100 %, 100 % v/v) and air-dried using hexamethyldisilane (HMDS; Sigma-Aldrich). Lastly, the samples were sputter-coated with gold before SEM analysis (EVO LS15, Zeiss, Oberkochen, Germany). SEM has been used previously as a method to assess the wear of tooth surfaces [29].

### 2.6 Statistical analysis

All statistical analyses were performed using GraphPad Prism version 8.1.0 for Windows, GraphPad Software, San Diego, California USA, www.graphpad.com. Statistics shown in the graphs were performed via one-way repeated measures ANOVA and post-hoc Tukey test (table shown in S1). Error bars show the standard deviation (SD).

## 3 Results

### 3.1 *P. gingivalis* survives in aerobic conditions in the presence of viable *C. albicans*

**Fig. 1:**
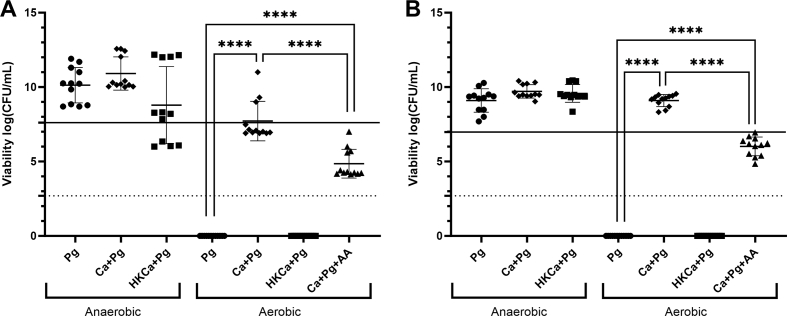
*P. gingivalis* survives in aerobic conditions only in the presence of *C. albicans* The effect of presence of *C. albicans* on viability in colony forming units per mL (CFU/mL) of *P. gingivalis* W83 (A) and ATCC 33277 (B) under aerobic and anaerobic conditions is shown. Under anaerobic conditions, *P. gingivalis* viability increases (above the solid line indicating the inoculation density), irrespective of the presence of *C. albicans*. Under aerobic conditions, *P. gingivalis* was undetectable (below dotted line indicating detection limit) when *C. albicans* was not present or heat-killed. The addition of Antimycin A inhibits the survival of *P. gingivalis* in the presence of *C. albicans.* Ca=*C. albicans*, Pg = *P. gingivalis*, HK = Heat-killed, AA = 10 μM Antimycin A. Each graph shows combined results of 3 independent experiments (p-value: **** = <0.0001).

To evaluate the influence of *C. albicans* on the growth of *P. gingivalis*, co-cultures were grown and viability of *P. gingivalis* was determined by CFU counting. For both *P. gingivalis* strains W83 and ATCC 33277, the presence of *C. albicans*, either viable or heat-killed, did not affect growth in anaerobic conditions compared to *P. gingivalis* alone (Fig. 1A and B). In contrast, aerobic conditions resulted in decreased viability of *P. gingivalis* as compared to anaerobic conditions. The CFU has decreased to below the detection limit, indicating bacterial cell death. The decrease in viability that occurred when culturing *P. gingivalis* alone aerobically compared to anaerobically is not occurring when viable *C. albicans* is present. This is seen for both *P. gingivalis* strains tested. This effect did not occur when in the presence of heat-killed *C. albicans*. In addition, viability was decreased by addition of Antimycin A, an inhibitor of oxidative phosphorylation, thereby reducing oxygen consumption by *C. albicans* [15]. *P. gingivalis* W83 showed lower viability in aerobic conditions compared to *P. gingivalis* ATCC 33277, when comparing the *P. gingivalis* cultured with live *C. albicans* to inoculation density (different for each strain, solid line in Fig. 1A and B). No differences in the growth of *C. albicans* were observed in the conditions where live *C. albicans* was present (data not shown).

### 3.2 The presence of living *C. albicans* increases gingipain production

**Fig. 2:**
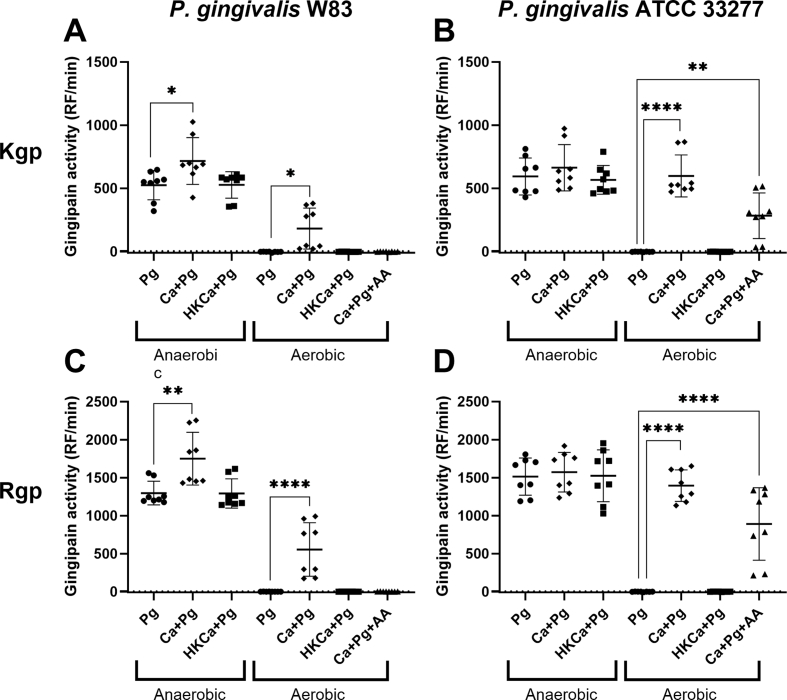
Degradation of fluorogenic substrates by *P. gingivalis* is increased in the presence of *C. albicans* Gingipain activity of Kgp (A, B) and Rgp (C, D) was observed in various conditions by *P. gingivalis* W83 (A, C) and ATCC 33277 (B, D). Degradation of the Kgp- and Rgp-specific substrates follow a similar trend. Under anaerobic conditions gingipain activity is high for all cultures. Only for W83 (A, C) gingipain activity increased in the presence of viable *C. albicans.* Under aerobic conditions there is only gingipain activity in the presence of viable *C. albicans.* When Antimycin A was added, there was a reduction of gingipain activity for ATCC 33277 (B, D) but absent for W83 (A, C). Ca=*C. albicans*, Pg = *P. gingivalis*, HK = Heat-killed, AA = 10 μM Antimycin A. The graphs show combined results of 4 independent experiments (p-values: * = <0.05; ** = <0.01; **** = <0.0001).

Following the finding that *C. albicans* facilitates the survival of *P. gingivalis* in aerobic conditions, it was investigated whether this also affected the virulence of *P. gingivalis.* As an indicator for virulence, the gingipain activity was determined by assessing the degradation of fluorogenic substrates specific for either Kgp or Rgp (Fig. 2). For strain W83 of *P. gingivalis*, gingipain activity was measured after culturing under anaerobic conditions, with a significant increase in activity when live *C. albicans* was added compared to *P. gingivalis* alone (Fig. 2A, C). Under aerobic conditions, there was only positive gingipain activity (above the threshold of 5 RF/min) in the presence of live *C. albicans*. These activities were noted for both Kgp and Rgp activity. For *P. gingivalis* strain ATCC 33277, comparable results have been found as of *P. gingivalis* W83 (Fig. 2B, D). One difference is that under aerobic conditions, the gingipain activity is still present upon addition of Antimycin A, which was not seen for W83. To summarize, the presence of *C. albicans* not only facilitates the survival of *P. gingivalis* under aerobic conditions, but the gingipain activity of *P. gingivalis* is also retained.

### 3.3 Als3 and Als1 mediate the adhesion of *P. gingivalis* to *C. albicans* hyphae

**Fig. 3:**
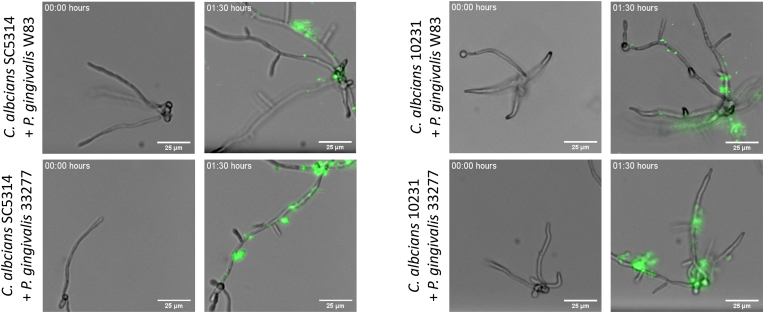
*P. gingivalis* adheres to hyphae of *C. albicans* Adherence of *P. gingivalis* labeled with carboxyfluorescein succinimidyl ester (CFSE) to C. *albicans* was observed in a Bioflux system by flowing *P. gingivalis* over the hyphae of *C. albicans*. *P. gingivalis* adheres to *C. albicans* hyphae within 1.5 h. Both strains of *P. gingivalis* (top: W83, bottom: ATCC 33277) adhered to both strains of *C. albicans* (left: SC5314, right: ATCC 10231). Scale bar indicates 25 μm in each picture.

The results of the metabolic analysis could be explained by a physical interaction between *P. gingivalis* and *C. albicans*. This was investigated using a dynamic flow system, used to study adherence of *P. gingivalis* to the hyphae of *C. albicans* over time. Adherence occurred within 1.5 h (Fig. 3, S2, S3). The bacterium appeared to cluster on the hyphae, with *P. gingivalis* ATCC 33277 showing this effect more predominantly than *P. gingivalis* W83 (Fig. 3). The hyphae of *C. albicans* ATCC 10231 were shorter than for *C. albicans* SC5314, but the level of *P. gingivalis* adherence was similar for the two *C. albicans* strains. Two strains of *C. albicans* were used to determine whether the length of the hyphae would have an influence on the ability of *P. gingivalis* to adhere, and to exclude the possibility that this effect is strain-specific.

**Fig. 4:**
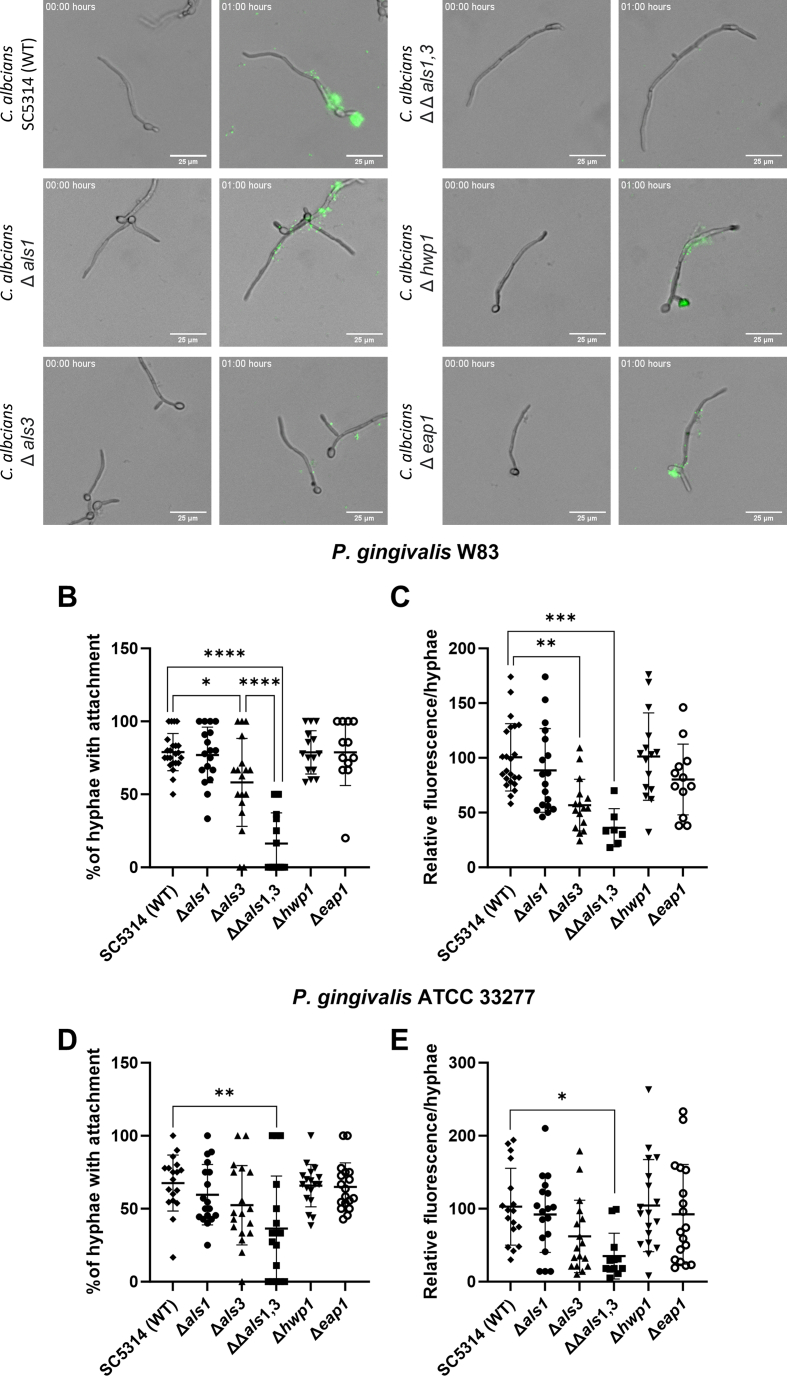
Als3 and Als1 are important for adhesion of *P. gingivalis* to hyphae of *C. albicans* Adherence of *P. gingivalis* labeled with carboxyfluorescein succinimidyl ester (CFSE) to various C. *albicans* mutants was observed in a Bioflux system by flowing the *P. gingivalis* over the hyphae of *C. albicans*. Mutants of Als1, Hwp1 and Eap1 showed adherence of *P. gingivalis*, as well as the wild type SC5314. Als3-deficient *C. albicans* had reduced adherence of *P. gingivalis* and for the double mutant Als1,3 even less adherence was observed. Scale bar in the images indicates 25 μm in each picture (A). The relative number of hyphae with fluorescently labeled *P. gingivalis* W83 (B) or ATCC 33277 (D) attached, relative to the total amount of hyphae per image. The relative mean fluorescent intensity per hyphae that had *P. gingivalis* W83 (C) or ATCC 33277 (E) attached, relative to the wild type *C. albicans* SC5314. Both strains of *P. gingivalis* show similar results. WT = Wild type. Each graph (B, C, D, E) shows combined results of 3 independent experiments (p-values: * = <0.05; ** = <0.01; *** = <0.001 **** = <0.0001).

In addition, the mechanism of adherence of *P. gingvialis* to *C. albicans* was investigated. Absence of cellwall proteins Als1, Hwp1 and Eap1 did not affect adherence of *P. gingivalis* (Fig. 4). For the Als3 mutant, the adherence was reduced and for the Als1,3 double mutant even less adherence was observed. For the Als1,3 double mutant, the amount of hyphae that had *P. gingivalis* attached to it was significantly reduced (Fig. 4B, D) compared to the wild type (WT, SC5314). This was the case for both strains of *P. gingivalis* (W83 and ATCC 33277). In addition, it was found that deletion of Als3 seemed to have a significant reduction on the amount of adhesion of *P. gingivalis* per hyphae and that this effect was increased when both Als1 and Als3 were absent (Fig. 4C, E). The variability in the measured values is much higher for *P. gingivalis* ATCC 33277 than for *P. gingivalis* W83.

### 3.4 Biofilm formation

**Fig. 5:**
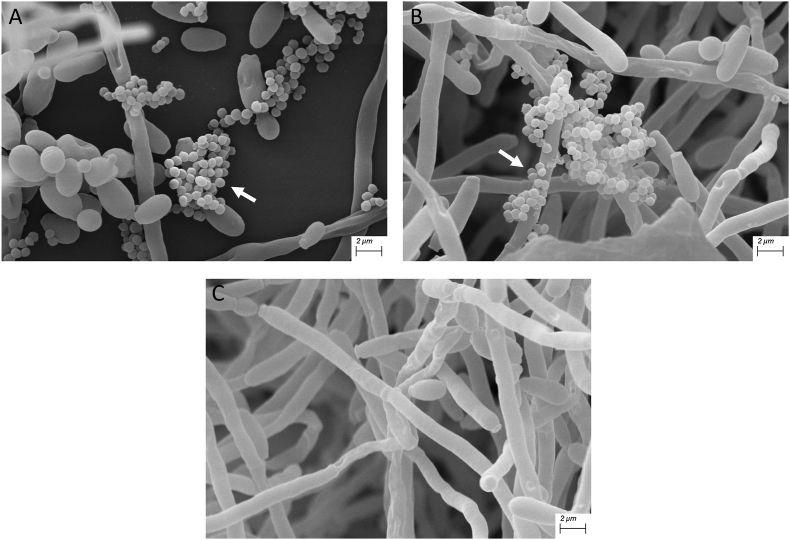
SEM images of biofilms containing both *C. albicans* and *P. gingivalis* Scanning electron microscopy (SEM) images of a biofilm containing both *C. albicans* (both yeast and hyphae) and *P. gingivalis* W83 (A) or ATCC 33277 (B) (small oval shaped bacteria, indicated by a white arrow). (C) Biofilm with only *C. albicans*. Scale bar indicates 2 μm in each picture.

To investigate the adhesion in more detail, mixed-species biofilms of *C. albicans* and *P. gingivalis* were grown on polystyrene surfaces to investigate the ability of *P. gingivalis* to attach to a *C. albicans* biofilm. SEM analysis show adherence of *P. gingivalis* W83 (Fig. 5A) and ATCC 33277 (Fig. 5B) to both yeast and hyphae of *C. albicans*. In Fig. 5A, it can also be seen that *P. gingivalis* adheres to each other, and not only to the hyphae. SEM images of a biofilm of only *C. albicans* is shown in Fig. 5C.

## 4 Discussion

The oral microbiome is a complex, diverse and dynamic biological system [1]. The microorganisms are constantly interacting with each other and with the host, which might influence the pathogenicity of certain species. The aim of this study was to characterize how the interaction between *P. gingivalis* and *C. albicans* could influence survival and virulence of *P. gingivalis* under aerobic conditions, conditions that prevail in the body of the host. When elucidating the mechanism of such interactions, these pathways of entry into the body could lead to a novel concept of the pathogenesis of *P. gingivalis* associated periodontitis. The current study has shown that the interaction of *P. gingivalis* with *C. albicans* allows for the anaerobic bacterium to survive in an aerobic environment. In addition, it is indicated that *P. gingivalis* retains its virulence, as shown by the activity of its secreted gingipains. There is a physical interaction between these two microbes which is mediated by the Als1 and Als3 proteins of *C. albicans,* where it can be suggested that Als1 is able to partially take over the function of Als3. In addition, these microorganisms can form mixed-species biofilms to which *P. gingivalis* can adhere and grow.

In a planktonic aerobic culture, it was found in the current study that *P. gingivalis* can only survive in the presence of live *C. albicans*. The explanation for this is that the survival of *P. gingivalis* is due to the oxygen consumption by *C. albicans*, which creates an environment with low oxygen levels in which *P. gingivalis* can survive and grow [15,30]. This same principle has been shown in previous research, for other anaerobic bacteria, *e.g. Bacteroides fragilis, Clostridium perfringens, Cutibacterium acnes,* and *Clostridioides difficile* (previously *Clostridium difficile*) [[31], [32], [33]]. Previous research by Bartnicka et al. has resulted in similar finding as the current study [30]. Both this previous research and the current study show that *C. albicans* can create a hypoxic environment to protect *P. gingivalis* against oxygen. It is postulated in that previous study that this is due to oxygen consumption by *C. albicans*. The current study adds on this by showing that inhibition of oxygen consumption by Antimycin A reduces the effect, confirming their hypothesis. This allows *C. albicans* to be a vector for anaerobic bacteria to survive and leads to the hypothesis that *C. albicans* could aid *P. gingivalis* to invade the host tissue [34].

One of the major virulence factors of *P. gingivalis* is the gingipain family of proteases. The measured gingipain activity showed a difference between the fluorogenic substrates used. This can be explained by the fact that both substrates contain an additional lysine residue, next to the 2 lysine or 2 arginine residues [27]. This means that the degradation of the Rgp-specific substrate is partially caused by the Kgp activity as well as the Rgp activity, meaning it is less specific for only Rgp. This explains why the gingipain activity of Rgp seems so much higher than Kgp. As the results of the two separate substrates show the same trend, it can be concluded that the different conditions in which *P. gingivalis* was cultured had similar effects on the activity or expression of both gingipains. In addition, the gingipain activity of the different cultures followed a similar trend as the survival of *P. gingivalis*, with two exceptions. First, it was found that for only *P. gingivalis* W83, the degradation of fluorogenic substrates by gingipains was increased in anaerobic conditions in the presence of live *C*. *albicans*. This effect was not due to increased number of *P. gingivalis* bacteria, as the number of viable bacteria were found to remain similar for all cultures in anaerobic conditions. This suggests that *C. albicans* had a positive influence on the production or activity of gingipains by *P. gingivalis*, although only for *P. gingivalis* W83. This increased gingipain activity in the presence of *C. albicans* has been shown before, but only for Rgp and not Kgp [35]. This indicates that the interaction with *C. albicans* can aid the bacterium in its pathogenicity. Whether this phenomenon also occurs in the oral cavity is unclear, as other microorganisms might also influence the gingipain activity by rendering the gingipains inactive. Second, *P. gingivalis* ATCC 33277 provides more gingipain activity in the presence of Antimycin A than *P. gingivalis* W83, which shows no gingipain activity at all under these conditions.

As mentioned before, it was found that *P. gingivalis* ATCC 33277 tended to aggregate more than *P. gingivalis* W83. This also explains the higher variability of the measured values for *P. gingivalis* ATCC 33277 when quantifying the adherence to *C. albicans* hyphae. The aggregation of this strain might have an influence on the fact that ATCC 33277 seems to be slightly more aerotolerant than W83. However, this specific phenomenon needs to be researched as there are no studies yet available that have investigated this. Another limitation of this study is that out-of-focus hyphae are difficult to trace, using the image analysis setup developed for this study. This might create bias in the data, and therefore it is not completely quantitative. However, a clear trend can be seen and the results of the analysis clearly correlate with the images provided.

The current study shows that adhesion for *P. gingivalis* is mediated by both Als3 and Als1 proteins of *C. albicans.* The absence of Als1 alone did not result in a reduction in adherence, but there was a significant difference in adherence when both Als1 and Als3 were absent. This suggests that Als1 is able to partially take over the function of Als3, with regard to binding to *P. gingivalis*. Previous research has shown that Als3 is involved in the mechanism adhesion of not only *P. gingivalis*, but also the adhesion of *Staphylococcus aureus* to hyphae of *C. albicans* [16,36]. However, in the current study it was found that Als1 was also involved in the mechanism of adhesion, which is something that has not been studied before for *P. gingivalis.* Other previous research has shown that the presence of *C. albicans* is necessary for *S. aureus* to disseminate to the bloodstream, and that this is likely mediated by macrophages that phagocytose the bacteria and relocate to the lymph nodes [37]. A similar mechanism might occur for *P. gingivalis*, as it was found in another study that *P. gingivalis* can survive within macrophages and exit them at a later stage [20]. However, the abundance of adhesion seems much higher in the case of *S. aureus* [16]*.* In addition, previous research found that *C. albicans* facilitates *P. gingivalis* invasion of gingival epithelial cells and fibroblasts [38]. More research is needed to confirm whether adhesion to *C. albicans* could also be a way for *P. gingivalis* to disseminate. Again, the study by Bartnicka et al. shows similar results as the current study, namely that the adhesion of *P. gingivalis* to *C. albicans* is mediated by Als3 [30]. The previous study used purified fungal proteins, whereas the current study used whole and live *C. albicans* cells. Observing the interaction between two microorganisms on a whole cell level gives a more complete view, while the approach of the previous research is limited to observing only one fungal protein at a time. This is shown in the current study, as it was found that Als1 is also involved in the interaction between *C. albicans* and *P. gingivalis*, in addition to Als3.

In the current study was found that adherence to the hyphae of *C. albicans* has a positive influence on the growth of *P. gingivalis*, but only for *P. gingivalis* W83. There have been studies before that have observed a mixed biofilm of *C. albicans* and *P. gingivalis* [30,36,39]. However, none have shown microscopic images using SEM where the adhesion of *P. gingivalis* to the hyphae of *C. albicans* is physically visible. In previous research, *P. gingivalis* has been observed as a rod-shaped bacterium [40]. However, in other studies, *P. gingivalis* has also been found as coccobacilli [41,42]. Adherence to *C. albicans* and the concentration of heme in the environment were found to an influence on *P. gingivalis* morphology [43].

*P. gingivalis* is mainly found in the gingival sulcus of people suffering from periodontitis, which is an environment with low oxygen [44]. Traditional treatment for periodontitis involves scaling and root planing, which disrupts the biofilm, potentially introducing oxygen into the environment. The current study has shown that the presence of *C. albicans* protects *P. gingivalis* against the oxygen, allowing it to survive, increasing its risk to invade the tissue and infiltrate the bloodstream. Of interest, similar studies on *C. albicans* and *Staphylococcus aureus* also indicated decreased susceptibility to antibiotics in co-cultures. This should be investigated in the future as antibiotic treatment following scaling and root planning is a common strategy [45].

In conclusion, this study shows that *P. gingivalis* survives and stays virulent in aerobic conditions when in the presence of *C. albicans*. The interaction with *C. albicans* could be a relevant mechanism for *P. gingivalis* to circumvent the aerobic environment of the host. Furthermore, it was found that Als1 and Als3 of *C. albicans* are involved in the interaction between the two microorganisms. This interaction leads to an increase in survival and virulence of *P. gingivalis* in oxygen-rich environment, which could be important for the pathogenicity of *P. gingivalis*. In addition, the adherence to *C. albicans* hyphae could provide an anchor point for *P. gingivalis* to colonize the oral cavity. These results emphasize the importance of interaction between different microbes in promoting survival, virulence and attachment of anaerobic pathogens. This could be essential in facilitating their penetration into the environment of the host.

## Funding details

This project is funded by the faculty of Dentistry, Academic Centre for Dentistry in Amsterdam, University of Amsterdam and Free University of Amsterdam.

## Disclosure statement

The authors declare that the research was conducted in the absence of any commercial or financial relationships that could be construed as a potential conflict of interest.

## CRediT authorship contribution statement

**Caroline A. de Jongh:** Conceptualization, Data curation, Formal analysis, Investigation, Methodology, Writing – original draft. **Floris J. Bikker:** Conceptualization, Funding acquisition, Supervision, Writing – review & editing. **Teun J. de Vries:** Conceptualization, Funding acquisition, Supervision, Writing – review & editing. **Arie Werner:** Methodology, Visualization, Writing – review & editing. **Susan Gibbs:** Conceptualization, Funding acquisition, Supervision, Writing – review & editing. **Bastiaan P. Krom:** Conceptualization, Funding acquisition, Supervision, Writing – review & editing.

## Declaration of competing interest

The authors declare that the research was conducted in the absence of any commercial or financial relationships that could be construed as a potential conflict of interest.

## Data Availability

Data will be made available on request.
